# Wetting of Dehydrated Hydrophilic *Pseudomonas fluorescens* Biofilms under the Action of External Body Forces

**DOI:** 10.1021/acs.langmuir.1c00528

**Published:** 2021-07-27

**Authors:** Michela Castigliano, Federica Recupido, Maria Petala, Margaritis Kostoglou, Sergio Caserta, Thodoris D. Karapantsios

**Affiliations:** † Department of Chemical, Materials and Industrial Production Engineering (DICMaPi), University of Naples Federico II, Piazzale V. Tecchio 80, 80125, Naples, Italy; ‡ Division of Chemical Technology, School of Chemistry, Aristotle University of Thessaloniki, University Box 116, 54 124 Thessaloniki, Greece; § Department of Civil Engineering, Aristotle University of Thessaloniki, 54 124 Thessaloniki, Greece; ∥ CEINGE Advanced Biotechnology, 80145 Naples, Italy

## Abstract

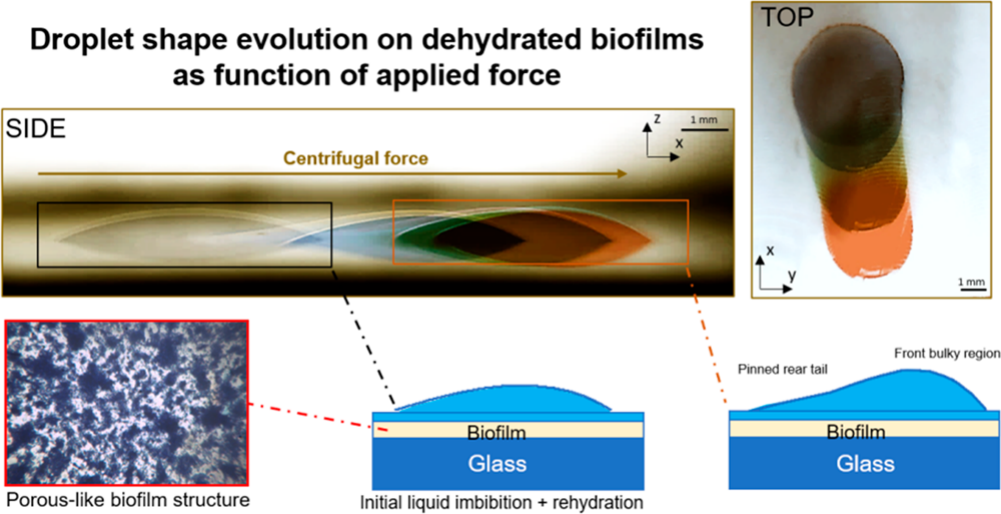

Wetting of dehydrated *Pseudomonas fluorescens* biofilms grown on glass substrates by an external liquid is employed as a means to investigate the complex morphology of these biofilms along with their capability to interact with external fluids. The porous structure left behind after dehydration induces interesting droplet spreading on the external surface and imbibition into pores upon wetting. Static contact angles and volume loss by imbibition measured right upon droplet deposition indicate that biofilms of higher incubation times show a higher porosity and effective hydrophilicity. Furthermore, during subsequent rotation tests, using Kerberos device, these properties dictate a peculiar forced wetting/spreading behavior. As rotation speed increases a long liquid tail forms progressively at the rear part of the droplet, which stays pinned at all times, while only the front part of the droplet depins and spreads. Interestingly, the experimentally determined retention force for the onset of droplet sliding on biofilm external surface is lower than that on pure glass. An effort is made to describe such complex forced wetting phenomena by presenting apparent contact angles, droplet length, droplet shape contours, and edges position as obtained from detailed image analysis.

## Introduction

Biofilms are communities of microorganisms enclosed by a matrix made of extra-cellular polymeric substances (EPS, a collective term describing polymeric substances such as proteins, polysaccharides, lipids, and extracellular DNA).^1−3^ Sessile microorganisms forming biofilms are markedly different from their planktonic counterparts (free-living cells suspended in the liquid bulk) in terms of gene expression, cellular physiology, and resistance against common antibiotics.^2^ Moreover, about 99% of the world’s bacterial population are found in the form of a biofilm at various stages of growth, showing different physical-chemical, morphological, and topographical properties according to the varieties of bacterial species.^4^ Bacteria form biofilms in both natural and industrial systems as a survival strategy. Fundamental understanding of biofilms represents strategic knowledge for public health, because of their strong resistance to antimicrobial agents. In industrial settings, biofilms are known for their ability to promote microbiofouling resulting in the clogging of hydraulic systems with consequent energy loss and possible cutbacks and shutdowns and in the reduction of conductive heat transfer across surfaces.^5^ In the case of the food industry, biofilms can contaminate processing equipment which are directly in contact with food, by also causing cross-contamination.^6^ However, biofilm formation is encouraged in some applications like in wastewater treatment^7^ and bioremediation processes.^8,9^

Biofilm formation is considered as a multistage process starting with microbial surface attachment and adhesion, followed by the production and accumulation of EPS that leads to the growth of sessile populations into mature biofilms and, eventually, concluded by cell detachment and dispersion.^10^ It is known that chemical (e.g., nutrient availability, temperature, pH fluctuations^4^) and mechanical stimuli (e.g., shear stress,^11−15^ electric or magnetic fields^13^ or gravity effect, i.e., surface curvature^16^) play a key role in biofilm development at different stages of growth, by regulating microbial transport, attachment on substrata and their subsequent adhesion, nutrients and oxygen delivery, and metabolites disposal through biofilm matrix and cell detachment.^17^ In addition, environmental conditions are also responsible for altering bacteria phenotype and genotype,^18,19^ bacterial quorum sensing,^20^ and morphology of biofilms.^21,22^

Wetting of biofilms by external liquids represents a largely unexplored research field.^23^ It must be stressed that bacterial adhesion, proliferation, and spreading over a surface to create a biofilm, which for brevity in literature is sometimes called wetting of biofilm, is different from the wetting of a biofilm by an external liquid that we deal with herein. In our case, the biofilm itself is the substrate on which wetting occurs. The latter shares several common features with the wetting of nonbiological porous layers by external liquids.^24−28^ It is recognized that biological surfaces are far from being ideal solid surfaces and as such experimentally measured quantities describing their wetting behavior represent apparent quantities, e.g., *apparent contact angles*.^29^ There are only a few systematic quantitative studies focusing on the physics of the wetting of biofilms by liquids and these studies refer mainly to *Bacillus subtilis* colonies grown on agar substrates.^30−32^ Attention to such biofilms stems from their extremely nonwettable character and the technological crucial interest in understanding how hydrophobicity can be reduced to remove deleterious biofilms by cleaning solutions.^30−32^ It was found that such biofilms are resistant toward water and nonpolar liquids,^31^ showing hydrophobic or even omniphobic behavior.^31,32^ Werb et al.^31^ and Falcón García et al.^32^ demonstrate that biofilm colonies show various wetting behavior depending on the selected system and conditions (such as bacterial strain, type of nutrient medium), i.e., from hydrophilic to superhydrophobic behavior. In this latter case, either lotus-like or rose petals-like wetting regimes are obtained; generally, the wetting behavior is strongly affected by the biofilm surface topography.^31^ The literature is rich on phenomena related to wetting of heterogeneous (not biological) substrates by droplets, where the effect of surface topography and chemical heterogeneities characteristic of the micropatterned surfaces have been experimentally and numerically investigated regarding the pinning/depinning behavior.^33−40^ The overall conclusions are that the presence of surface heterogeneity affects the contact angle hysteresis and when external forces are employed, overcoming pinning is strongly influenced by the nature of microscale defects,^33^ which act like energy barriers, hampering droplet motion.^36^ Interestingly, it is found that, in the case of the wetting of liquids on tilted patterned surfaces, the critical Bond number, seen as the dimensionless force needed to initiate droplet sliding, is strongly affected by the shape and spatial arrangements of surface patterning as shown in refs (36,38, and 39), whereas, close to the critical Bond number, jerky motion of the droplet is observed as a consequence of stick–slips dynamics. Furthermore, according to the wettability nature of surface heterogeneities, droplet depinning can be deferred either by hydrophobic patterns, which hinder droplets at the front, inducing droplet compression and height increase, or by hydrophilic ones, which hold a droplet on its back side, inducing severe elongation and reduction of its height. These aspects are of interest for the case of hydrophilic dehydrated biofilms that are studied herein.

Wetting of dehydrated hydrophilic biofilms by external liquids constitutes an interesting but very complex case. Contact angles of sessile droplets on hydrophilic microbial lawns, after an initial dehydration step, have frequently been used in physicochemical models as a measure of microbial cell surface structure.^41^ The wetting of such biofilms is seen as a powerful tool to investigate the complexity of biofilm morphology and its capability to interact with external liquids.^23^ This is so because wetting of such biofilms is accompanied by liquid imbibition (capillarity-driven flow in the pores left behind after dehydration), simultaneously with (re)hydration of dried solid matter. Even in the absence of solid mass rehydration, wetting together with imbibition is a quite complex process where several wetting states can be distinguished depending on the applied conditions. For instance, the Cassie–Baxter impregnating wetting state corresponds to the classical imbibition (normal and lateral) into the porous layer.^42^ Moreover, wetting and imbibition by a finite volume sessile droplet over a thin dry porous layer is different from the cases where either the liquid comes from an infinite liquid reservoir or the droplet lies on a thick dry porous layer.^41^ When studying the wetting of dehydrated hydrophilic biofilms by liquids, the concomitant imbibition and solid mass rehydration processes imply that the standard definition of thermodynamic wetting arising between liquid and solid interfaces, is difficult to realize and the phenomenology of biofilm wetting is quite complex to interpret. To our best knowledge, Recupido et al.,^43^ were the first to examine *static (equilibrium) wetting* properties on dehydrated hydrophilic biofilms of *Pseudomonas fluorescens* AR 11 cultivated on real (non-nutrient) substrates, i.e., glass coupons, under different flow conditions. Biofilms grown at different conditions exhibit different internal structure and morphology which need be known to allow strategies either for their effective removal from surfaces if they represent a contamination (“bad” biofilms) or for intensification of their development if they represent a means for nutrients production (“good” biofilms). To do so, hydrophilic biofilms are dehydrated to leave behind a solid porous structure which then must be analyzed. These authors found that wetting properties do not vary significatively as a function of the different biofilm morphology, induced by growing them at different flow conditions. Their dehydrated biofilms showed both hydrophilic and oleophilic behavior. To expand^43^ in investigating biofilm morphology effects, it is fundamental to also consider the application of external body forces on sessile droplets (forced wetting), which in principle may be hydrodynamic, electrical, or mechanical ones and can drive the system beyond thermodynamic equilibrium. As far as we know, there is no evidence in the literature about forced wetting on dehydrated hydrophilic biofilms.

In this work, *Pseudomonas fluorescens* AR 11 bacteria cultivated on glass substrates is used as a model microorganism to form biofilms. Wetting properties on dehydrated biofilms are investigated using a custom-made device, named Kerberos, dedicated to the study of physics of forced wetting of droplets deposited on different substrates.^44−47^ Kerberos employs independent control of centrifugal and gravitational forces acting on droplets by combining rotation and tilting while at the same time monitoring the droplet shape deformation and motion by three cameras viewing the droplet at the three orthogonal directions (*X*, *Y*, and *Z*). The present experiments refer exclusively to rotation of horizontal substrates (no tilting). For porous/micropatterned surfaces like biofilms, it is well-known that wettability is strongly affected by surface properties such as morphology, which, however, is driven by the different biofilm growth conditions i.e., nutrient composition, shear flow, or incubation time. The goal of this study is to identify basic features of the forced wetting phenomena of dehydrated porous biofilms up to the onset of droplet sliding as a tool to examine biofilm morphology and describe an appropriate image analysis scheme for data extraction and evaluation. The investigation of the droplet contact angles as well as of depinning thresholds during rotation tests, is devoted to estimate the retention forces needed for droplet onset for sliding, which actually represent an indirect measure of biofilm surface adhesion.

## Experimental Section

### Microorganism and Culture Conditions

Lyophilized *Pseudomonas fluorescens* AR 11 bacteria are provided by DSMZ-German Collection of Microorganisms and Cell Cultures (Braunschweig, Germany) and reactivated according to the manufacturer protocol.^48^ The rehydration is performed in 0.5 mL of liquid complex medium (5 g/L of peptone from animal tissue and 3 g/L of meat extract of double-distilled water) and subsequent mechanical agitation for 30 min. The bacterial suspension is grown at 30 °C overnight at 80 rpm and then diluted to achieve an optical density (OD_600 nm_) ≈ 0.5 cm^–1^, which corresponds to around 10^7^ cells/mL. Biofilms are developed using M9 minimal medium containing the following constituents, while pH is adjusted at 7:6.8 g/L of Na_2_HPO_4_, 3 g/L of KH_2_PO_4_, 0.5 g/L of NaCl, and 1 g/L NH_4_Cl, 0.24 g/L MgSO_4_·7H_2_O, 0.04 g/L CaCl_2_·2H_2_O, 0.05 g/L EDTA, 8.3 mg/L FeCl_3_, 0.84 mg/L ZnCl_2_, 0.1 mg/L CuCl_2_·2 H_2_O, 0.1 mg/L CoCl_2_·2H_2_O, 0.1 mg/L H_3_BO_3_, and 0.016 mg/L MnCl_2_·4 H_2_O and 0.1% glucose.

### Experimental Setup

Biofilm formation and growth is conducted under stagnant fluid conditions. Biofilms are formed on microscope slides (76.2 × 25.4 × 1 mm^3^) placed inside polystyrene Petri-dishes (Φ = 90 mm) (PROMED, Italy). Glass coupons are cleaned with commercial anionic surfactant P1, rinsed with tap water, then immersed in 95% ethanol solution, rinsed again with distilled water, and finally dried at 200 °C for 2 h. In each Petri dish, a microscope slide is placed horizontally and covered with 1.5 mL of bacterial suspension and 13.5 mL of sterile minimal medium (1:10 dilution). The resulting overall thickness of the fluid layer is 2.3 mm. Biofilms are formed on both sides of the glass substrate. The total area of the glass is 38.7 cm^2^, and the total area of the Petri dish exposed to the medium is 70.22 cm^2^. The selected operative conditions are meant to ensure aerobic conditions for bacterial growth. Biofilm formation and development is carried out at 30 °C for 7 days. Nutrient replacement is performed after 24 h from the inoculation and then, every 48 h, in order to promote bacterial viability. The evolution of glucose concentration within bacterial suspensions is estimated during the first 24 h after inoculation and displayed in S1 (Supporting Information, SI).

### Planktonic and Biofilm Growth

Planktonic growth is monitored via spectrophotometric measurements. The turbidity of the microbial suspensions is measured as OD_600 nm_, which is an indirect measure of the cell density (i.e., number of cells per unit of volume). Before OD_600 nm_ measurement, bacterial suspensions are gently homogenized and then 2 mL are sampled and measured. Biofilm growth is determined by scratching biofilm from glass surfaces using a sterile toothbrush and resuspending it in 15 mL of sterile M9 phosphate solution. First, the OD_600 nm_ turbidity of the mechanically removed and resuspended biofilm is measured. Then, the Total Organic Carbon (TOC) and Dissolved Organic Carbon (DOC) of the resuspended scratched sample are also determined aiming to quantify the biofilm organic matter. TOC is measured by using a TOC analyzer (TOC 5000 A, Shimazu, Japan). TOC measurements are obtained indirectly by subtracting the Inorganic Carbon (IC) from the Total Carbon (TC) as reported by ref (49). Biofilm DOC is measured, after samples are filtered through 0.2 μm-membrane. For each test, sampling is performed after 3 and 7 days, respectively.

### Optical Microscopy

Biofilm coverage of glass slides is monitored by optical microscopy, using an upright microscope (Leitz Wetzlar Diaplan, Germany). Images are acquired using a 40×/0.70 PL Fluotar objective. In order to increase the contrast between the biofilm covered surfaces and the surrounding blank glass area, glass substrates are stained with Crystal Violet solution (0.1% in bidistilled water). Briefly, 3 mL of Crystal Violet solution is added to each sample. Samples are incubated for 10 min. Rinsing with bidistilled water is performed to remove the excess of Crystal Violet solution. At each incubation time, the morphology of three independent glass samples is examined, by acquiring, for each sample, images at three independent positions. Biofilm coverage area over the total area of the image (%) is evaluated using the commercial software Image Pro Plus 6.1 (Media Cybernetics, U.S.A.). Covered area normalized with respect to the entire image size is considered as an estimate of biofilm coverage of the glass sample. Measurements of averaged covered area% are reported for different incubation times, Standard Deviation (SD) provides an estimate of samples variability.

### Forced Wetting Tests

**Figure 1:**
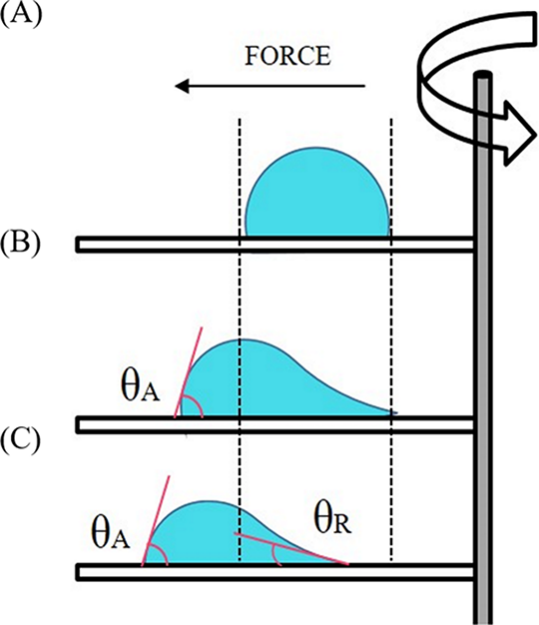
Sessile droplet side-view at different rotation speed over a clean smooth surface. (A) static droplet when no rotation is employed, (B) spreading of droplet with the front edge moving at the advancing contact angle whereas the rear edge remains pinned, and (C) Sliding of droplet with the front edge moving at the advancing contact angle and the rear edge moving at the receding contact angle.

**Figure 2:**
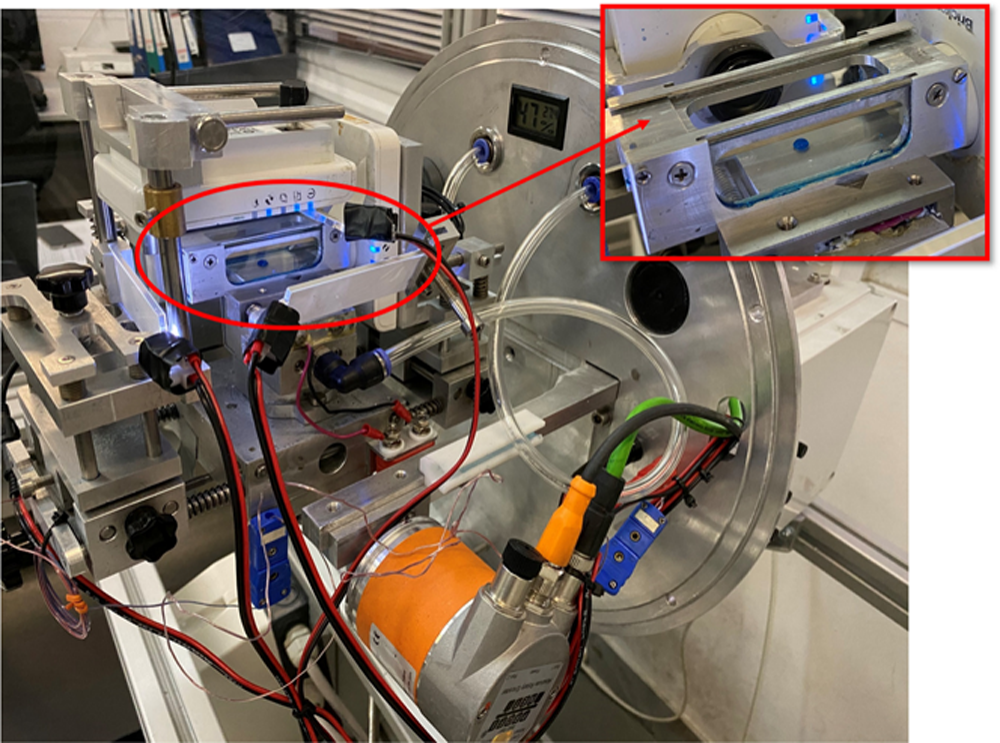
Overview of Kerberos rotating unit together with the test cell. Inset: Close-up of the test cell.

Forced wetting tests on biofilm-coated surfaces are carried out using Kerberos, an innovative device whose major functionality is the study of the evolution of sessile droplet shape and motion under external body forces as shown in previous research works.^44−47^ The device combines simultaneous rotation and tilting to provide independent control of tangential and normal forces acting on a droplet placed on a solid substrate. Kerberos allows monitoring of the 3D droplet shape as a function of time, by recording images with three independent wireless cameras that observe the droplet simultaneously along the 3 orthogonal directions *X*, *Y*, *Z*. Cameras rotate and tilt together with the sample. In particular top, side, and back views visualize the droplet on the (*x*,*y*), (*x*,*z*), and (*y*,*z*) plane, respectively. During an experiment, the chamber containing the sample and the cameras allows to control the temperature and air humidity surrounding the sample. Detailed description of the main features and capabilities of the device along with analysis of spreading/sliding of droplets on different solid substrates at varying tilting angles and rotation speeds can be found in.^45,47^ In this work no tilting is done, while centrifugal forces are employed to induce droplet deformation, spreading, and sliding depending on the applied conditions (Figure 1). Tests are carried out at 25 ± 2 °C and at RH = 50 ± 5%. Biofilm-coated glass substrates are first allowed to drain by gravity for 20 min and then are dehydrated under continuous N_2_ flow to avoid sample oxidation. This is done inside a sealed custom-made Plexiglas chamber, reported in ref (50). The chamber is equipped with inlet and outlet tubes having valves for regulating gas flow. Sample dehydration is performed at room temperature for 2 h, i.e., the time necessary to achieve stable sample weight after dehydration of the very thin biofilms. Subsequently, wettability tests are carried out using water droplets colored by a “Brillant Blue” dye solution (0.5 g/L, Hina Dye Chem Industries, China) in bidistilled water, in order to enhance the contrast of droplet images with their background. Wetting properties of biofilms are examined using 10 μL droplets and by programming Kerberos to reach a target rotation speed, RS, of 100 rpm with an increase rate of 1 rpm/s. The range 0–100 rpm/s corresponds to centrifugal accelerations, *α*_c_, in the range 0–27.4 m/s^2^. These values are calculated as *α*_c_ = ω^2^ · *r* with ω = 2*πf* and *f* = *RS*/60 where ω is the angular speed of rotation and *r* (0.25m) is the distance between the droplet and the axis of rotation. It is noted that for the employed experimental configuration *α*_c_ takes a value equal to the gravitational acceleration at about 60 rpm. Wetting experiments of water solution on the uncoated glass (no biofilm) under the application of centrifugal forces are used as negative controls. During tests, biofilm coated and blank glass substrates are placed inside a test cell (82 × 30 × 22 mm^3^, shown in Figure 2) which is essentially a parallelepiped aluminum frame accommodating glass windows between edges in all sides. The chamber is meant to minimize the amount of air surrounding the droplet and also keep it at stable temperature/humidity and therefore suppress droplet evaporation. The test cell also permits avoiding direct exposure of the three cameras to the biological samples. Droplet deposition is done using a 500 μL syringe (1750 LTN SYR, Hamilton) equipped with a dispenser (PB600-1, Hamilton, step 1/50 of syringe volume) that delivers a fixed liquid volume of 10 μL. The three cameras (WCB-100A, Brickcom) equipped by 7× magnification lens (Olloclip, 3 in 1 Lens) record droplet shape with a frame rate of 10 fps. Videos acquired from the cameras are stored in *avi* format to be processed.

### Automatic Matlab Code

Droplet videos are processed by using a custom-made Matlab code (v. 2013) described in detail in ref (46). The code delivers the geometrical parameters of the droplet, as viewed from the side and the top, versus time. Once the user provides some input parameters (such as the region of interest surrounding the droplet, and the baseline that represents the projection of the substrate), the software analyses automatically a series of video images and from them it reconstructs the droplet shape contour during the rotation test, and estimates a number of geometrical parameters. In this study, only the droplet front and rear apparent contact angles, the contact length and the location of droplet edges with respect to time, (i.e., with respect to instantaneous rotation speed), are utilized. Droplet contour (perimeter) coordinates are also obtained as output for each value of the rotation speed. To scale measurements from pixel to mm a calibration is carefully performed independently for the side and top views, in order to calculate adequate conversion factors.

### Image Intensity Profile

Apart from the aforementioned Matlab code (which runs without possibility for interventions), detailed analysis of droplet images is conducted to identify the complex wetting behavior of biofilm-coated surfaces. Every frame is converted from RGB to gray scale and analyzed using a commercial image analysis software (Image Pro Plus 6.1). Image analysis measurements include image-histograms, (reporting the percentage of pixels of the image as a function of the gray intensity); and linear intensity profiles (reporting gray intensity along a diametral segment). These tools are used to identify droplet edges and, therefore, the distribution of water along sample. Alternative visualization of droplet edges is also obtained by applying edge detection filters (Sobel), based on the calculation of intensity gradient profiles.^51^ Although Image Pro Plus software requires more effort by the user compared to the automatic Matlab code, it allows versatility in selecting the background for the determination of droplet contour even at the level of single frames which cannot be provided by the automatic Matlab code.

## Results and Discussion

### Planktonic and Biofilm Growth

In the present work, biofilms are grown on glass substrates under stagnant fluid conditions at two incubation times: 3 and 7 days, respectively. In Table 1, planktonic and biofilm growth are reported in terms of turbidity of planktonic and attached cells and of TOC and DOC of the resuspended biofilms as a function of the incubation time. Measurements are conducted before replacing the nutrient medium. The analysis of planktonic growth shows an increase of OD_600 nm_, due to the medium replacement and thus to the restoration of the initial conditions, which suggests the achievement of a good physiological state of the planktonic cells, typical of the exponential phase of bacterial growth, since OD_600 nm_ is lower than 1 cm^–1^.^52^ Biofilm OD_600 nm_ represents an indirect measure of the whole biofilm mass, which increases during the testing period.^53^ Biofilm TOC and DOC also increase over time. This implies that, at higher incubation times biofilm formation is enhanced, primarily due to the food replacement. More specifically, it is found that the TOC measured at 7 days, is about 5 times higher with respect to the value measured at 3 days, while the DOC value is about 4 times higher. It is important to point out that biofilm TOC refers to the overall available organic matter within the biofilm structure, which includes both the part contained in bacterial cells and that contained in the dissolved matter surrounding the cells. DOC corresponds exclusively to dissolved organic carbon that is predominantly found in EPS; therefore, this parameter represents a quantitative estimation of biofilm EPS. The observed increase in the difference between TOC and DOC with the incubation time, indicates a progressive increase of the number of bacterial cells attached to biofilm.

**Table 1:** Planktonic and Biofilm Growth as a Function of the Incubation Time in Terms of Turbidity of the Planktonic and Resuspended Attached Cells and of TOC/DOC of the Attached Cells^a^

incubation time (days)	planktonic OD_600 nm_ (cm^–1^)	biofilm OD_600 nm_ (cm^–1^)	biofilm TOC (mg/L)	biofilm DOC (mg/L)
0	0.067 ± 0.0057	(−)	(−)	(−)
3	0.637 ± 0.0036	0.0207 ± 0.0005	10.77 ± 0.48	9.10 ± 1.47
7	0.879 ± 0.0145	0.1047 ± 0.0007	49.04 ± 3.88	39.14 ± 5.8

a Data are reported as average ± standard deviation, measured from three independent replicates.

### Biofilm Morphology

**Figure 3:**
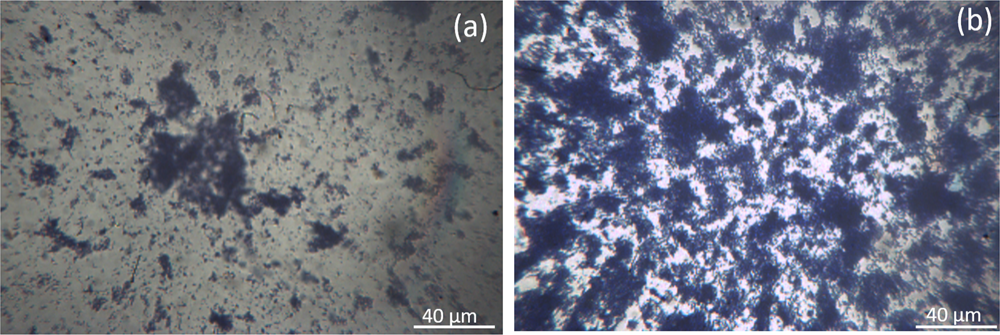
Optical microscopy images of biofilms vs incubation time. Biofilms are cultivated in stagnant fluid conditions for (a) 3 days and (b) 7 days. Biofilms are labeled with 0.1% Crystal Violet solution. 40× magnification, scale bar = 40 μm. Gray scale images are processed by a smoothing filter to minimize light nonuniformity and background noise and segmented in order to measure the biofilm covered area.

Under stagnant fluid conditions, biofilms are in the form of patchy like clusters consisting of rod-shaped bacteria cells and EPS, surrounded by empty spaces (Figure 3). Cells/EPS clusters appear denser and thicker at longer incubation time. The surface% covered by biofilms is measured by analyzing optical images stained with crystal violet. Table 2 reports the characterization of the biofilm coverage area as a function of incubation time. The biofilm coverage area rises from 5.7% to 34.78% when the incubation time is extended from 3 to 7 days. However, under the examined conditions samples present relatively high heterogeneity, as measured by standard deviations calculated from three independent measurements. This may be associated with inhomogeneities in biofilm wetting, as will be pointed out in biofilm wetting properties section.

**Table 2:** Biofilm Coverage Area (%) vs Incubation Time^a^

incubation time (days)	coverage area ± SD (%)
3	5.75 ± 1.56
7	34.78 ± 8.76

a Data are reported as average and standard deviations from three independent measurements.

### Biofilm Wetting Properties

**Figure 4:**
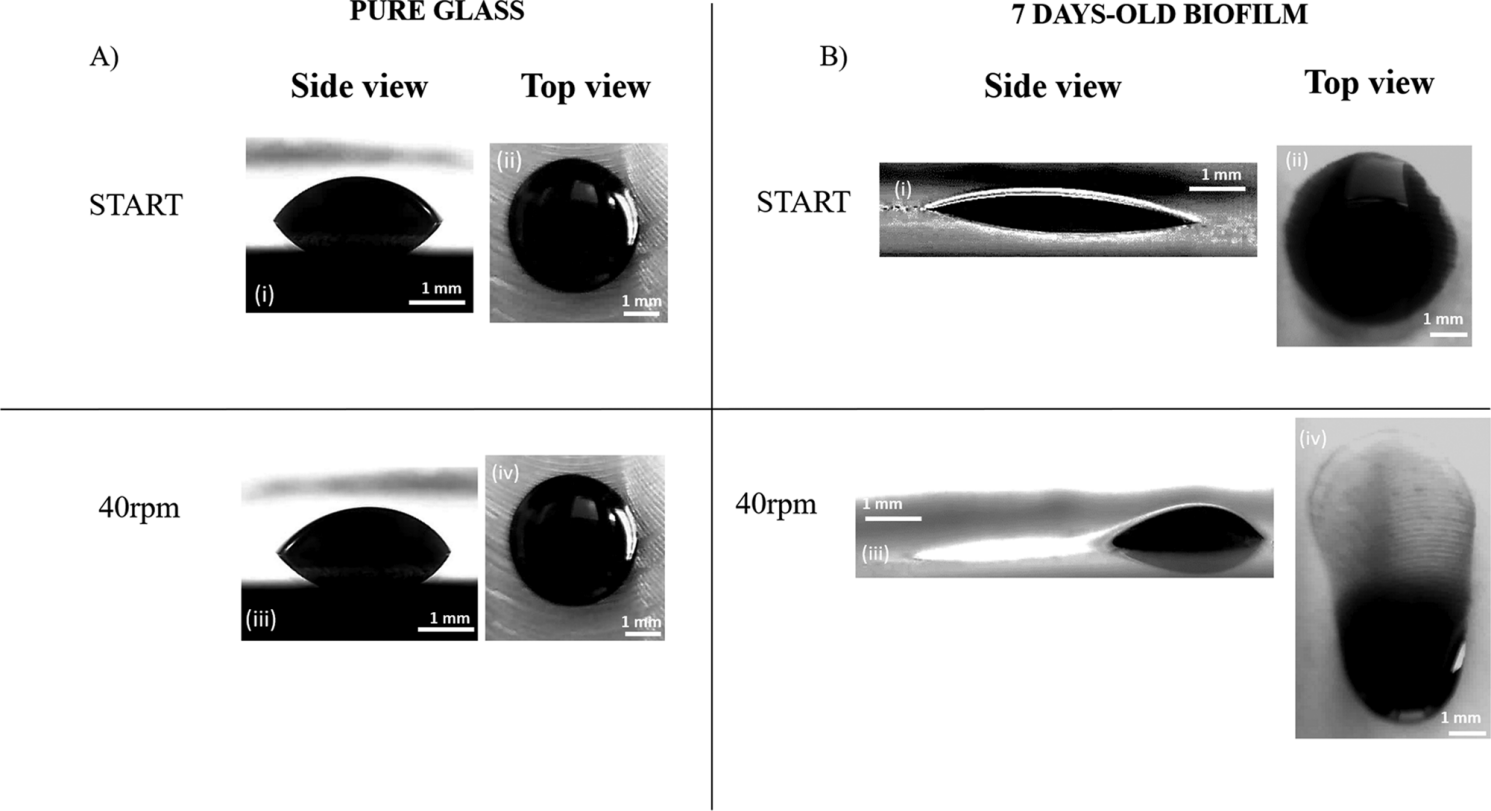
Side and top views of 10 μL droplets placed onto glass coupons at the start of the rotation test, 0 rpm, and at 40 rpm. Rotation speed increases at a rate of 1 rpm/s. Scale bar = 1 mm. Centrifugal forces make the droplet move from left to right in the images. (A) Pure glass (uncoated substrate) and (B) 7 days-old biofilm coated glass slide.

Wetting experiments are carried out under the application of centrifugal forces (Kerberos) on glass substrates coated with biofilms cultivated in stagnant fluid conditions at two different incubation times: 3 days and 7 days, respectively. Experiments are performed with the rotation speed increasing from 0 to a 100 rpm following a ramp with a slope of 1 rpm/s. Before presenting detailed information on how apparent contact angles, contact lengths, droplet contours, and droplet edge positions vary with rotation speed, a few representative droplet images are displayed to allow describing some qualitative features of wetting of dehydrated biofilms. This is done in Figure 4 where images taken at two specific rotation speeds are shown: 0 rpm (beginning of the experiment is designated as START), and 40 rpm. In Figure 4, side (i–iii) and top (ii, iv) views of droplets placed onto uncoated glass substrates (Figure 4A) and on 7 days-old-biofilm-coated glass substrates (Figure 4B), are compared. At the moment of droplet deposition on the biofilm coated substrate, droplets exhibit spontaneous fast spreading, followed by the evidence of imbibition effects due to capillary forces related to the porosity of the dehydrated biofilm layer. More specifically, shortly after droplet deposition, we observe fast droplet spreading corresponding to droplet length increase and contact angle decrease. Afterward, droplet imbibition (at constant length and at decreasing contact angle) occurs. This is typical for droplets placed on any hydrophilic porous layer and leads to decreased contact angles due to volume loss in the drop caused by the imbibition.^25^ This preliminary observation might indicate that the biofilm-coated surfaces show a higher effective hydrophilicity compared to pure glass. However, the possibility exists that part of the droplet edges get pinned soon after deposition because of local heterogeneities and defects of the biofilm surface. In such case, the observed progressively lower contact angles might be also the result of volume reduction due to imbibition, at least down to an angle where depinning occurs. This is in line with other studies related to contact line pinning on heterogeneous substrates.^54−57^ These observations are qualitatively comparable with the results obtained by Recupido et al.^43^ but are opposite to the case of *B. subtilis* colonies grown on Agar substrates,^30−32^ which show different wetting behavior i.e., from hydrophilic to superhydrophobic depending on growth conditions. Droplets gently deposited on uncoated glass show always sharp circular edges, typical of a spherical cap (Figure 4 A,i–ii). On the contrary, droplets on biofilm-coated surfaces at the end of spontaneous spreading (at the time scale of the present observations) show irregular and jagged edges (Figure 4B,i-ii). In earlier experiments with Kerberos,^45^ when a 10 μL droplet was placed on a plain uncoated glass substrate it was observed that the critical value to induce droplet spreading (elongation of its front edge while its rear edge remains pinned) was 54 ± 2 rpm. On the present biofilm-coated substrates, already at 40 rpm the droplet moves. This suggests that the force needed for the inception of droplet motion is lower compared to the case of pure glass. It is probable that due to imbibition a thin moist biofilm layer surrounds the droplet which facilitates motion. This is in line with the notion of capillary forces continuing to elongate the droplet, although at a much lower speed, after its initial fast spreading upon deposition.

During motion of a droplet onto the biofilm, its rear edge never depins from its original site but instead a liquid tail develops at the rear side of the droplet, where a translucent residual moist layer seems to be left on the biofilm surface (Figure 4B,iii–iv). Such a tail never appears on uncoated pure glass. It is possible that once the biofilm gets rehydrated it obstructs depinning because of its hydrophilic nature. Because of nondepinning of the droplet rear edge, droplets on dehydrated hydrophilic biofilms are seen only to spread (only front edge moving) and not to slide (both edges moving). Furthermore, as the droplet spreads under the influence of centrifugal force its bulk liquid squeezes toward its front side yielding a dark droplet region with a smaller base (contact length) and larger height. As a result, larger apparent contact angles are observed at both edges of this dark droplet region. This is also never seen on uncoated pure glass substrates. Consequently, during droplet motion the liquid volume is distributed unevenly along the droplet length, identifying two different regions: a front bulky dark region, where most of the liquid is present, and a long thin white tail at the droplet’s back, where the liquid thickness seems negligible, (Figure 4Biii–iv). This anomalous wetting behavior, related to the porous nature of the dehydrated biofilm layer, reflects on the definition of the droplet contact angle. As already mentioned, the angle measured by direct observation of the images should be more appropriately considered as an apparent contact angle, the real 3-phase contact line between liquid, gas, and the surface being hidden within the surface pores. The measured values of the rear apparent contact angle depend on whether image analysis is applied to the bulky front dark region or to the long thin white tail.

**Figure 5:**
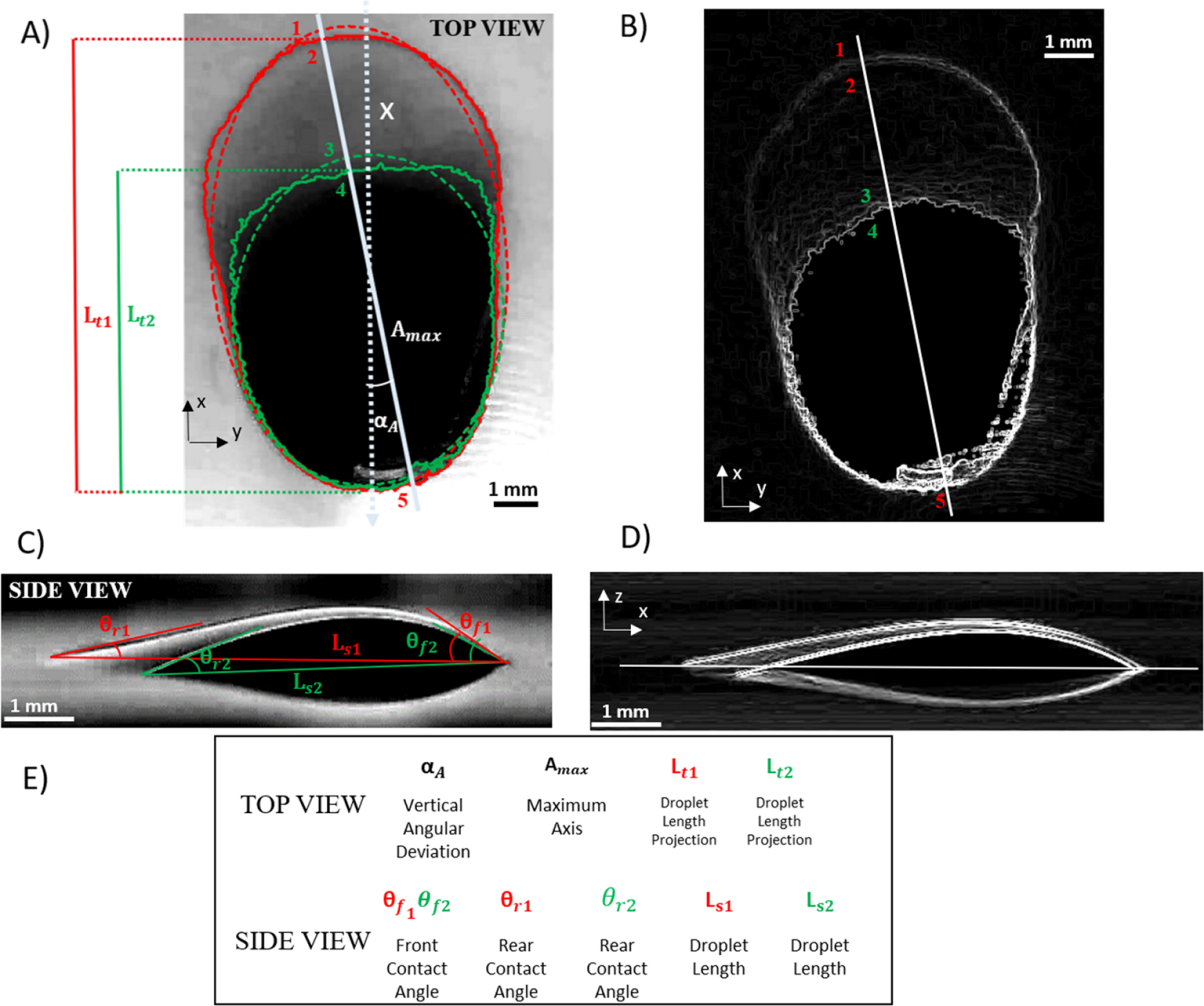
(A) Top view of 10 μL droplet placed onto a biofilm after 3 days of incubation, during rotation test. The image is detected at the rotation speed of 50 rpm. The red and green contours represent the real edges (solid lines) and the ellipse approximation (dashed lines), for both the dark bulky and the whole droplet region including the thin tail, respectively. Scale bar = 1 mm. (B) same image as (A) processed with the edge detection Sobel filter. (C) Side view of the same 10 μL droplet shown in (A). The two lines (red and green) represent the droplet length of the dark bulky region (*L*_s2_) and of the whole droplet (*L*_s1_), respectively. In red and green, the front (θ_f1_ and θ_f2_) and rear (θ_r1_ and θ_r2_) contact angles of the droplet versus the corresponding contact lines for the dark and white regions in image (C). (D) same image as (C) processed with the edge detection Sobel filter. (E) Summary of the 2D wetting parameters obtained from both top (image A) and side (image C) views with their definitions, symbols, and colors.

**Figure 6:**
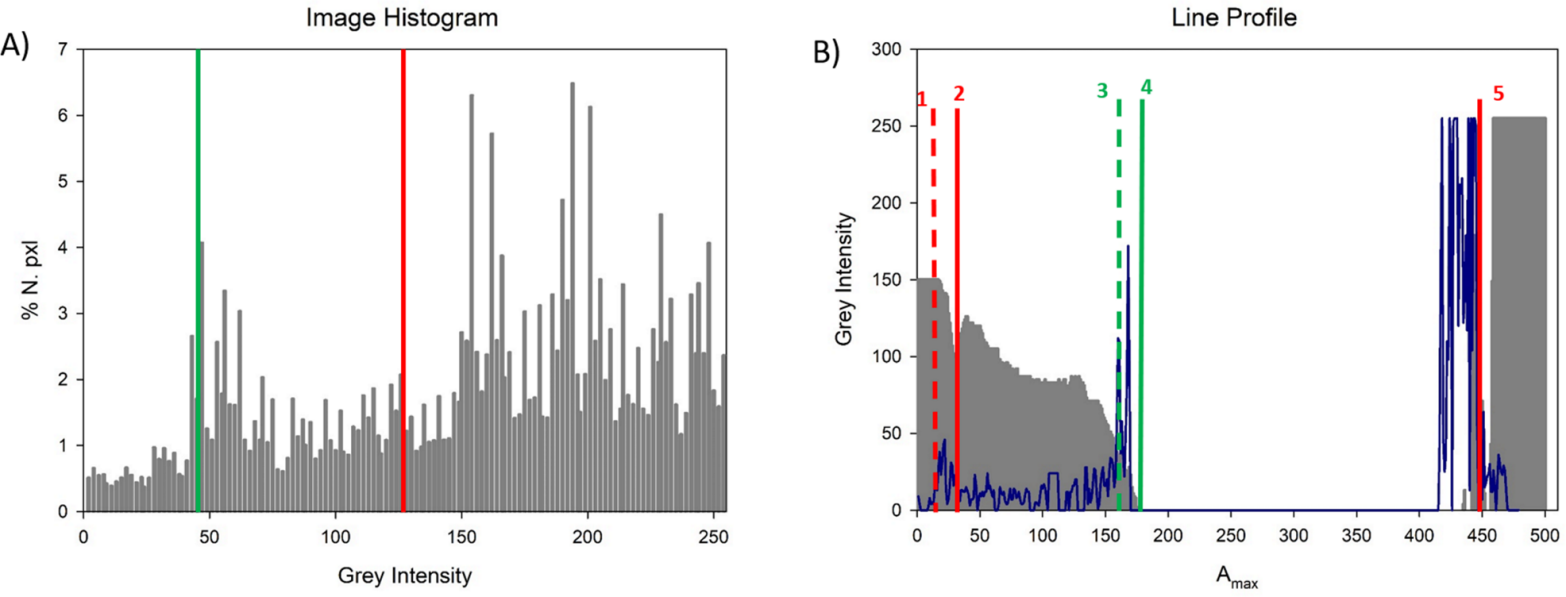
(A) Histogram of the gray intensity profile for the top view of Figure 5. Gray scale in 8-bit monochrome images ranges from 0 (black) to 255 (white). (A). Two thresholds (45 and 125) are selected to define the two droplet contours. Threshold values can be variable over different frames of the same experiment. (B) Gray intensity profile along the maximum axis of the droplet (white line, Figure 5A) matched with the Sobel filter intensity profile along the same axis. Positions 1 and 2 represent the edges of the whole droplet surface (including the thin tail) for the ellipsoid approximation and the jagged contour, respectively. Positions 3 and 4 refer to the edges of the dark bulky region of the droplet, again for the ellipsoid approximation and the jagged contour, respectively; finally, point 5 indicates the univocal definition of the droplet front edge, where the intensity profile sharply rises to the base value.

In Figure 5A,C, the top and side view of a droplet spreading on a biofilm-coated glass under centrifugal forces is displayed. To identify the edges of the drop, images are processed using a Sobel filter^57^ that calculates local light intensity gradients along the image plane. Objects edges, corresponding to maxima in the gray gradients, are visible as bright lines. Sobel filtered images of top and side views are reported in Figure 5B,D, together with a white line representing the droplet major axis, *A*_max_. Image histogram of top view in Figure 5A is reported in Figure 6A. By defining two threshold values (indicated by the green and red lines, respectively), it is possible to segment the droplet edges, identifying the edges of the dark bulky region (green) and of the entire droplet area including the thin white tail (red).

Gray intensity profile of the image in Figure 5A along the major axis of the ellipse/droplet *A*_max_ is reported as the gray area in Figure 6B, and compared with the same measurement done on the Sobel filtered image (Figure 5B), reported as a blue line. Following the line profile, it is possible to identify five positions, reported by numbers 1 to 5 in both Figures 5 and 6 (see captions for details). The intensity profile shows a decreasing trend when moving from the droplet back (1) to front (5). In particular, the light intensity converges to 0 over the entire dark region (between 4 and 5). This detailed analysis brings important information about the effective distribution of the liquid over the surface during the movement of the droplet, where the gray intensity can be considered as directly related to the amount of water covering the surface. The distribution of liquid on the surface is a nontrivial question in the case of complex surfaces, where the wetting phenomena are complicated by the porosity and nonuniformity of the dehydrated biofilm.

The identification of droplet edges along the maximum axis (*A*_max_, Figure 5A,B) and of the angular deviation with respect to the *x* axis (*α*_A_) shows the projection of droplet length along the moving direction (*x*), which is the quantity typically measured as contact length from the side view. The observed angular deviation *α*_A_ with respect to the *x* direction, is the result of a minor imperfection in leveling the substrate with respect to the horizontal because of which the gravitational acceleration creates a lateral force component affecting the direction of droplet motion or due to heterogeneities and micropatterns of biofilms surface, which may create preferential paths in droplet motion. This minor discrepancy is not relevant for the scopes of our work. The two measurements of the droplet length obtained from the top view, identified by the red and green edges, are defined as *L*_T__1_ and *L*_T__2_ and refer to the whole droplet and the dark bulky region, respectively. It is worth mentioning that the difference between jagged (continuous) and ellipsoidal (dashed) edges lead to a less than 2% discrepancy in the estimated droplet area and a less than 10% discrepancy in the estimated droplet length. In our analysis we refer to top lengths *L*_*T*__1_ and *L*_*T*__2_, as measured by the ellipsoidal edges. By observing the droplet from the side (Figure 5C), it is possible to clearly identify the dark bulky region at the front, and the thin long tail at the back of the moving liquid. This also leads to distinct definitions of the front and rear contact angles as well as of the droplet length. Two front contact angles, *θ*_f__1_ and *θ*_f__2_ can be defined as the angles formed by the solid surface and the tangent to the droplet profile at the front edge, for the whole droplet surface (red edge) and for the dark bulky region (green edge), respectively. Similarly, two different rear contact angles are defined as *θ*_r__1_ and *θ*_r__2_. Additionally, two droplet lengths are measured from side images defined as *L*_s__1_ and *L*_s__2_. The definition of two droplet lengths implies the existence of two different baselines for the dark bulky region and for the whole droplet, respectively (shown in Figure 5C in green and red colors). It is worth mentioning that the two baselines are measured independently, and do not superimpose, but can differ by a few degrees (about 5°). This discrepancy is due to the optical effect related to the complex light reflection on the uneven water interface and due to the fact that the red rear edge is slightly further from the camera with respect to the green rear edge. The temporal evolution of the side contour is evaluated during the rotation test. In the case of the dark bulky region (green edge), contours and relevant geometrical parameters can be obtained automatically by the Matlab code reported in ref (46). The red edges of the entire droplet, including the long thin tail, require (at this stage) a time-consuming manual analysis, since droplet translucent edges are hardly identified by automatic algorithms. In our results, we compare measurements obtained manually for both green and red edges, with the one obtained automatically in the case of green edges. A summary of the parameters measured from top and side images is reported in Figure 5E.

**Figure 7:**
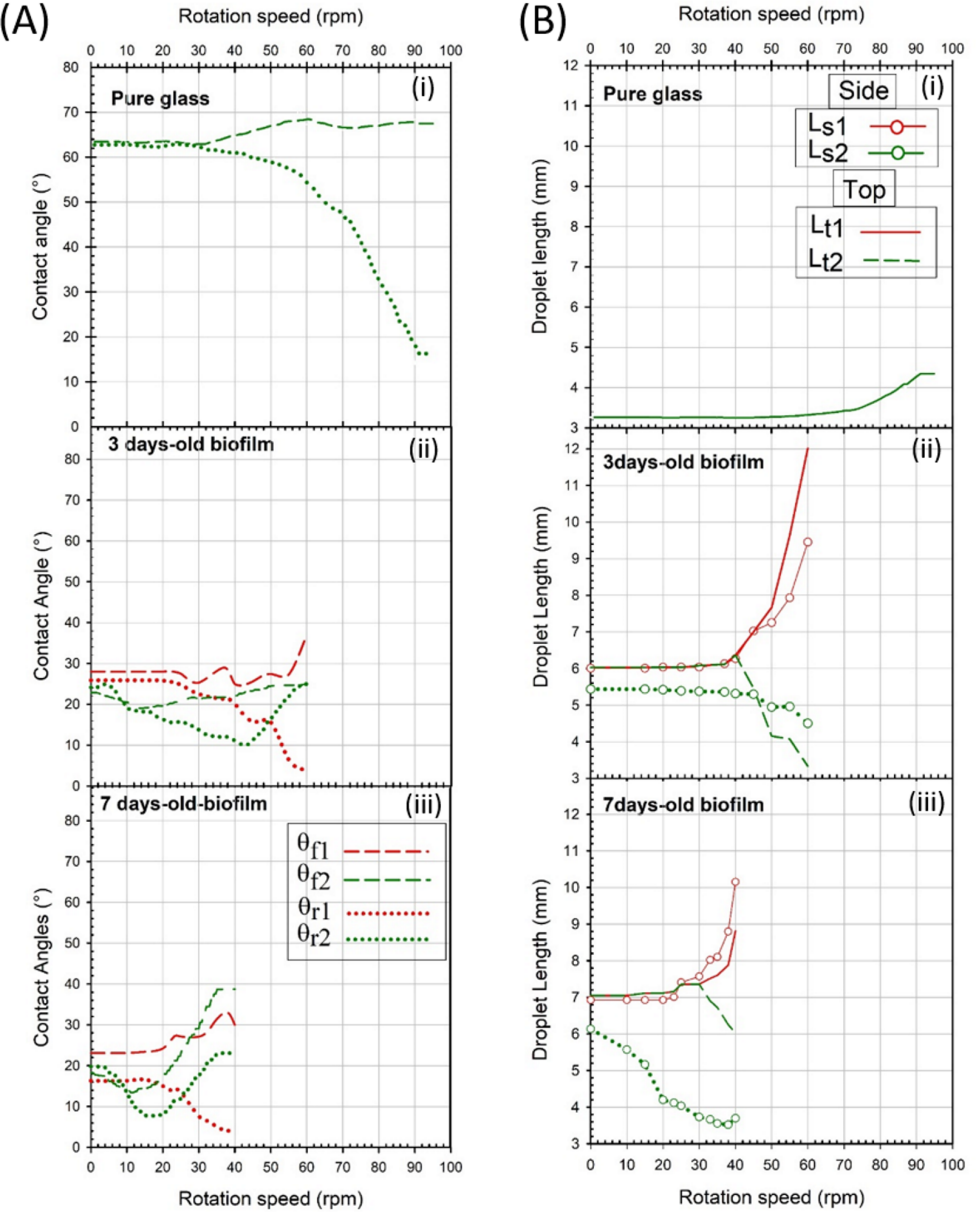
(A) Front and rear contact angles vs rotation speed, (B) Droplet length vs rotation speed evaluated from either top or side views for 10 μL droplets on biofilms cultivated with different incubation times. Rotation speed is increased up to 100 rpm with a speed rate of 1 rpm/s. (i) Pure glass (uncoated substrate), (ii) 3 days-old biofilm, and (iii) 7 days-old biofilm. Detailed description of the measured parameters is reported in Figure 5E. Briefly: *θ*_f__1_ and *θ*_f__2_ are the front contact angles of the dark bulky region and the whole droplet, respectively. *θ*_r__1_ and *θ*_r__2_ are the receding contact angles of the dark bulky region and the whole droplet, respectively. *L*_*s*__1_ and *L*_*s*__2_ are the droplet lengths, evaluated from side view, of the dark bulky region and the whole droplet, respectively. *L*_t__1_ and *L*_t__2_ are the droplet lengths, evaluated from top view, of the dark bulky region and the whole droplet, respectively.

The above-described analysis is applied systematically, to obtain detailed information on forced wetting on biofilm coated surfaces, by using the Kerberos device. Front and rear contact angles (Figure 7A) and droplet length (Figure 7B) are reported as a function of rotation speed for 10 μL droplet on different samples: (i) pure glass; (ii) 3-days old biofilm; (iii) 7-days old biofilm, respectively. Contact angles and droplet length profiles vs rotation speed in Figure 7 refer to an indicative experiment selected among the performed repetitions. This is done, instead of estimating average values, because biofilms are natural entities with highly uneven surfaces and so they are never the same even if they grow at the same conditions. Figure S2 presents repeatability runs to show the degree of variability among experiments. Experimental data are shown after smoothing using a mild running average function to minimize spikes. For pure glass, as observed in Figure 4, the entire droplet is always observed as a dark bulky region defined by a clearly visible unique interface. This means that there is no difference between the measurements obtained by the automatic Matlab code and the Image Pro Plus analysis. Results show that droplets are initially axisymmetric (front and rear contact angles are similar) and, by increasing the rotation speed, spreading/sliding behavior is observed. Once the rotation speed reaches a critical value (about 30 rpm) the shape of the droplet starts to lean (i.e., deform along the force direction) without any effect on the droplet length. This is seen by the changes in the front and rear contact angles (Figure 7A,i). As the rotation speed increases, the front contact angle reaches the value of the advancing contact angle, (∼67° at about 55 rpm) where spreading takes place, i.e., the front edge starts moving and so the droplet length increases (Figure 7B,i). During spreading, the rear edge remains pinned but the rear contact angle progressively decreases. As the rotation speed increases further, and while the front edge continues to move at the advancing contact angle value, a speed is reached where the rear contact angle (Figure 7A,i, green dotted line) attains the receding contact angle value (∼11° at about 90 rpm). At this moment, the rear edge depins and the droplets start sliding together and as a result the droplet length remains constant. (Figure 7B,i). The obtained results are in good agreement with those reported in ref (45). The difference between the present rotation speed for the onset of sliding (90 rpm) and the corresponding rotation speed in that study (70 rpm) is justified by the different advancing contact angle values, i.e., ∼67° here and ∼55° in that study, in applying the Furmidge relation^45^ for the estimation of the force for the onset of sliding. Such differences in contact angle values are not uncommon when employing different cleaning procedures. In the lower plots of Figure 7 the aforementioned wetting analysis is repeated for surfaces covered by 3- and 7-days old dehydrated biofilms, (Figure 7A,B,ii–iii), respectively. In these plots, side and top view data are presented from image analysis of both the entire droplet contour (red lines) and the dark bulky droplet contour (green lines). Side and top view data on droplet length are in good agreement (average deviation less than 5%). There is a discrepancy between different color contours chiefly related to contrast variations that affect measurements. Lower initial contact angles are observed on biofilms with respect to the pure glass substrate, as also reported by ref (43) for the case of static wetting. This is partly because of the imbibition of droplet liquid into the pores of the dehydrated biofilms which renders them effectively more hydrophilic. The hydrophilic behavior of biofilm is further confirmed by the fact that the value of contact angle is lower for longer incubation times, corresponding to a thicker biofilm with a higher surface coverage (34.78%, see Table 2). Regarding the whole droplet surface (droplet red contour), the presence of the narrow transparent boundary around the droplet disturbs the measurement of the initial contact angles. Despite this difficulty, it seems that contact angles do not change up to about 20 rpm. When the rotation speed exceeds 20 rpm then the front contact angle, *θ*_f__1_ (of the whole droplet region) increases along with the rotation speed (Figure 7A,ii–iii, dashed red lines), whereas, the rear contact angle, *θ*_r__1_, (Figure 7A, ii and iii, dotted red lines) reduces. For the 3-days old biofilm, for speeds up to about 40 rpm this change in contact angles is associated exclusively with a change in droplet shape which gradually shifts from axisymmetric to a tear-like shape. For speeds above 40 rpm the droplet spreads gradually (front edge moves) until at around 60 rpm fast spreading occurs and the droplet disappears from the field of view. During all this activity, the rear edge of the droplet never depins producing a long thin tail and a severe droplet elongation as rotation speed increases. For the 7-days old biofilm, spreading starts already at about 20 rpm whereas at 40 rpm the droplet disappears swiftly from the cameras view with its rear edge still pinned at its original location. It is interesting that as the rotation speed increases the rear white side of the droplet gets progressively very narrow and transparent with the rear contact angles attaining very small values (less than 5°). This means that at the moment of disappearance from the cameras the thin tail of the droplet soaks entirely into the pores of the biofilm. The fast spreading at high rpm values in both biofilms is reflected at the abrupt rise of the droplet length at the last stages of the experiment (Figure 7B,ii–iii). The rotation speed for droplet departure in the 7-day biofilm is clearly lower than the departure speed in the 3-day biofilm indicating a more hydrophilic behavior of the more mature biofilm. The dark bulky droplet region containing most of the liquid (droplet green contour) also behaves in a strange way (Figure 7A,B,ii–iii). Almost right from the onset of rotation both the front and rear contact angles become smaller than their initial values. This is because some liquid is forced to imbibe into the pores (liquid volume is “lost”). At some moderate rotation speed (∼10 rpm in both biofilms), liquid squeezes toward the front edges and as a result the front contact angles increase. In the 3-days biofilm, the rear edge of the whole droplet region remains pinned and so the rear contact angle decreases until it becomes 10° at about 45 rpm From that speed on, the rear edge of the dark region depins and the dark droplet region itself gradually squeezes toward the front edges yielding a smaller dark droplet length. At 60 rpm the droplet suddenly runs off the field of view. In the 7-days biofilm, the rear edge depins at a much lower speed (∼15 rpm) and so the dark droplet region starts earlier to squeeze and eventually at 40 rpm it abruptly escapes the scene. Summing up, at longer incubation times (7-days versus 3-days) the initial contact angles are a bit lower and the initial droplet length is a bit higher owing to the thicker biofilm which offers more pores for liquid imbibition. At a quick glance, these quantities indicate that the 7-days biofilm is more hydrophilic than the 3-days biofilm. The same argument also holds for the retention force (proportional to rotation speed) needed for droplet runoff which is lower for the 7-days biofilm. It is important to remember, however, that the observed contact angles on the dehydrated biofilms can be only considered as apparent quantities. Inasmuch as we are aware, such biofilm wetting behavior is reported for the first time in literature and is attributed to the simultaneous imbibition phenomena occurring in the porous structure of the dehydrated hydrophilic biofilms.

**Figure 8:**
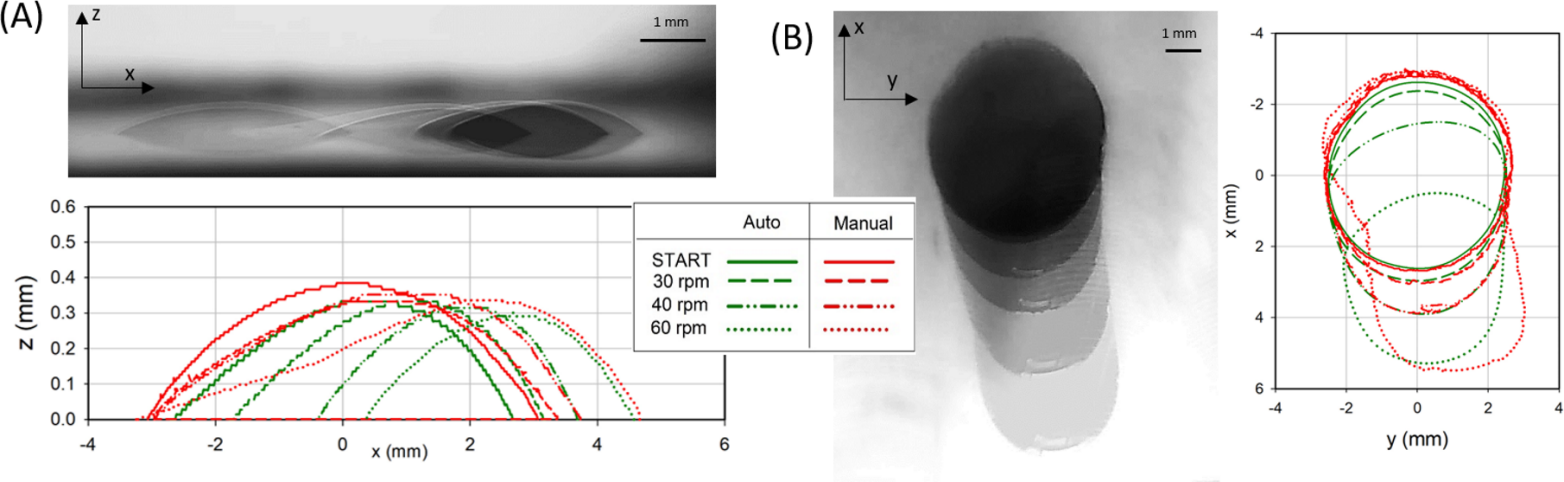
(A) side view (up) and (B) top view overlays (left) of a 10 μL droplet placed onto 3 days-old biofilm during rotation test. For each overlay, side and top contour evolution as a function of the rotation speed (low and right) are reported. Outlines detected either manually and automatically are displayed for both top and side views.

Figure 8 shows for both side (A) and top (B) views the droplet contour evolution during the application of centrifugal force, supported by images overlays. For clarity, contours are shown at representative frames (rotation speeds). Colored overlays of both views are shown in S3 of the SI. Experimental side and top contours are detected for both the entire droplet (red lines) and the dark bulky droplet region (green lines) mentioned before. For the dark bulky region (Figure 8, green lines), the movement of the front and rear edges as rotation speed increases, induces an apparent sliding of this dark bulky region of the drop, that contains most of the liquid mass. As a consequence, the green contour appears to shrink along the centrifugal direction. On the contrary, the advancement of front edges and the pinning of rear edges for the entire droplet contours leads to a very elongated droplet that only spreads (front edge moves) and never slides (rear edge always pinned) (Figure 8, red lines), due to the initial wicking on porous biofilm systems. Figure 8 allows one to appreciate the actual dimensions of the droplet. Side contours reveal that the droplet at its most shrinked state has a length about 10 times larger than its height, and this goes up to 20 times at its most elongated state. Top views demonstrate that droplet contours are quite smooth and symmetric despite the stochastic morphology of the dehydrated biofilms. This implies that heterogeneities in the biofilm morphology are at a much smaller scale than the size of the droplet which means that droplets of this size are suitable to study the macroscopic wetting properties of these biofilms. This is even more so since the shape of the elongated red contours appreciably resemble those reported on clean glass substrates.^45^

**Figure 9:**
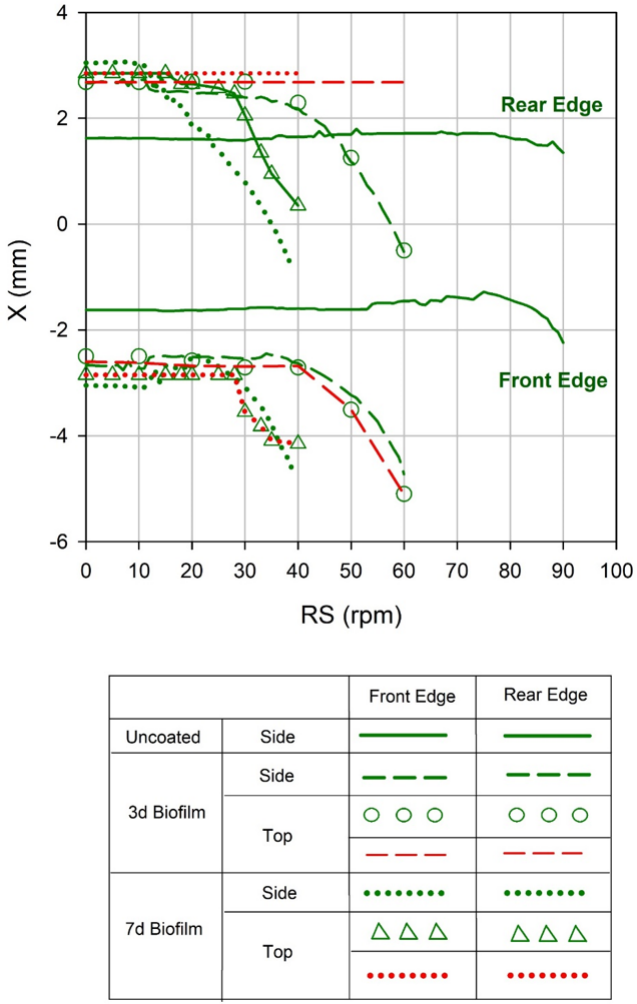
*X* front and rear positions of 10 μL droplet on pure glass and on 3- and 7- days old biofilms as a function of the rotation speed. *X* positions are detected from both side and top views.

The position of the front and the rear droplet edges are also derived from the above measurements. Figure 9 displays the front and rear positions along the centrifugal direction (*x*) of the droplet on 3-days old and 7-days old biofilms, respectively. As with Figures 7 and 8, the highly uneven nature of biofilms even if they are grown under the same conditions make us present data from a representative droplet for each of the examined growth conditions. Both manually and automatically calculated data are reported. Details about the reproducibility of our measurements can be found in Figure S2. The motion of the droplet edges gives additional information with respect to the droplet length evolution by revealing the pinning behavior of the droplet and allowing better identification of spreading and sliding. The edge positions are calculated from the top and side views, for both the entire droplet and the dark bulky droplet region (reported in Figure 9 as red and green contours, respectively). For pure glass, *x*-edges position shows a constant trend until sliding occurs. In the case of biofilm, front and rear *x*-edges evaluated from side views are approximately matched with the ones detected from the top view. For the dark bulky region, the front and the rear edges start moving at lower rotation speeds, the longer is the incubation time, but for each incubation time the two front edges move approximately at the same moment. In both the 3- and 7-days case the rear edges as detected from the long tail (red curves) never move during the entire rotation test, corroborating the above-mentioned results reported in Figure 8.

## Conclusions

In this work, wetting properties of dehydrated hydrophilic biofilms by an external liquid under the application of centrifugal forces are examined for the first time. A specialized device, Kerberos, capable of studying the wetting properties of sessile droplets under the action of body forces, i.e., gravitational and centrifugal ones, is employed. Biofilms are cultivated in stagnant fluid conditions at two incubation times: 3 and 7 days, respectively. An accurate analysis of wetting properties is conducted by means of an automatic and a manual computer code that combines fast treatment of long videos with the versatility in resolving contrast problems of single frames. It is found that the porous structure left behind after dehydration leads to simultaneous wetting and imbibition of the biofilm by the liquid of the droplet. This peculiar behavior affects the droplet shape right upon its deposition on the biofilm surface leading to small apparent contact angles as a result of liquid being sucked into the pores. As a result, dehydrated porous biofilms when compared to bare glass appear effectively more hydrophilic in the sense that contact angles on the biofilm surface are lower because of the volume loss by imbibition. This effective hydrophilicity increases at higher incubation times because the biofilm is thicker with more pores. Furthermore, during rotation tests the rear edge of the droplet never depins leading to the formation of a severely elongated droplet with a long thin tail at its back. This is attributed to imbibition which keeps the liquid sucked in the pores at all times. All in all, forced wetting tests reveal features of the dehydrated biofilm morphology that dictate their interaction with external fluids and so can be valuable for a wide range of applications, including biofilm removal or prevention i.e., optimization of detergent solution formulations or design of antimicrobial/antibiofouling surfaces impairing microbial attachment.

## Supporting Information Available

Figure S1, Measurement of C-source concentration within bacterial suspensions; Figure S2, repeatability check of front and rear contact angles and droplet length of 10 μL droplets on 3 days-old and 7 days-old biofilms; and Figure S3, colored overlays from side and top views of 10 μL droplet on 3 days-old-biofilm during rotation test (PDF)

## Abbreviations

EPS: Extra Polymeric Substances
TC: Total Carbon
IC: Inorganic Carbon
TOC: Total Organic Carbon
DOC: Dissolved Organic Carbon
SD: Standard Deviation
RS: Rotation Speed

## References

[ref1] Stoodley P. Biofilms: Flow Disrupts Communication. Nat. Microbiol. 2016, 1, 15012 10.1038/nmicrobiol.2015.12. 27571757

[ref2] Hall-Stoodley L.; Costerton J. W.; Stoodley P. Bacterial Biofilms: From the Natural Environment to Infectious Diseases. Nat. Rev. Microbiol. 2004, 2, 95–108. 10.1038/nrmicro821. 15040259

[ref3] Carniello V.; Peterson B. W.; van der Mei H. C.; Busscher H. J. Physico-chemistry from initial bacterial adhesion to surface-programmed biofilm growth. Adv. Colloid Interface Sci. 2018, 261, 1–14. 10.1016/j.cis.2018.10.005. 30376953

[ref4] Garrett T. G.; Bhakoo M.; Zhang Z. Bacterial Adhesion and Biofilms on Surfaces. Prog. Nat. Sci. 2008, 18, 1049–56. 10.1016/j.pnsc.2008.04.001.

[ref5] Rao Toleti S. Microbial Fouling and Corrosion: Fundamentals and Mechanisms, In Operational and Environmental Consequences of Large Industrial Cooling Water Systems; Springer: Boston, 2012; Vol. 4, pp 95–126.

[ref6] Marchand S.; De Block J.; De Jonghe V.; Coorevits A.; Heyndrickx M.; Herman L. Biofilm Formation in Milk Production and Processing Environments; Influence on Milk Quality and Safety. Compr. Rev. Food Sci. Food Saf. 2012, 11, 133–143.

[ref7] Andersson S.; Kuttuva Rajarao G.; Land C. J.; Dalhammar G. Biofilm formation and interactions of bacterial strains found in wastewater treatment systems. FEMS Microbiol. Lett. 2008, 283, 83–90. 10.1111/j.1574-6968.2008.01149.x. 18422628

[ref8] Cycoń M.; Piotrowska-Seget Z. Pyrethroid-degrading microorganisms and their potential for the bioremediation of contaminated soils: a review. Front. Microbiol. 2016, 7, 1463 10.3389/fmicb.2016.01463. 27695449 PMC5023672

[ref9] Azeredo J.; Azevedo N. F.; Briandet R.; Cerca N.; Coenye T.; Costa A. R.; Desvaux M.; Di Bonaventura G.; Hebraud M.; Jaglic Z.; Kacaniova M.; Knøchel S.; Lourenco A.; Mergulhao F.; Meyer R. L.; Nychas G.; Simoes M.; Tresse O.; Sternberg C. Critical Review on Biofilm methods. Crit. Rev. Microbiol. 2017, 43, 313–351. 10.1080/1040841X.2016.1208146. 27868469

[ref10] Flemming H.; Wingender J. The biofilm matrix. Nat. Rev. Microbiol. 2010, 8, 623–633. 10.1038/nrmicro2415. 20676145

[ref11] Zhang X.; Yuan H.; Wang Y.; Guan L.; Zeng Z.; Jiang Z.; Zhang X. Cell Surface Energy Affects the Structure of Microalgal Biofilm. Langmuir 2020, 36, 3057–3063. 10.1021/acs.langmuir.0c00274. 32160744

[ref12] Zheng S.; Bawazir M.; Dhall A.; Kim H. E.; He L.; Heo J.; Hwang G. Implications of Surface properties, bacterial motility and hydrodynamic conditions on bacterial sensing and their initial adhesion. Front. Bioeng. Biotechnol. 2021, 9, 1–22. 10.3389/fbioe.2021.643722. PMC7907602 33644027

[ref13] Persat A.; Nadell C. D.; Kim M. K.; Ingremeau F.; Siryaporn A.; Drescher K.; Wingreen N. S.; Bassler B. L.; Gitai Z.; Stone H. A. The mechanical word of bacteria. Cell 2015, 161, 988–997. 10.1016/j.cell.2015.05.005. 26000479 PMC4451180

[ref14] Zarabadi M. P.; Paquet-Mercier F.; Charette S. J.; Greener J. Hydrodynamic Effects on Biofilms at the Biointerface Using a Microfluidic Electrochemical Cell: Case Study of *Pseudomonas sp*. Langmuir 2017, 33, 2041–2049. 10.1021/acs.langmuir.6b03889. 28147485

[ref15] Krsmanovic M.; Biswas D.; Ali H.; Kumar A.; Ghosh R.; Dickerson A. K. Hydrodynamics and surface properties influence biofilm proliferation. Adv. Colloid Interface Sci. 2021, 288, 102336 10.1016/j.cis.2020.102336. 33421727

[ref16] Chang Y. W.; Fragkopoulos A. A.; Marquez S. M.; Kim H. D.; Angelini T. E.; Fernández-Nieves A. Biofilm formation in geometries with different surface curvature and oxygen availability. New J. Phys. 2015, 17, 033017 10.1088/1367-2630/17/3/033017.

[ref17] Toyofuku M.; Inaba T.; Kiyokawa T.; Obana N.; Yawata Y.; Nomura N. Environmental factors that shape biofilm formation. Biosci., Biotechnol., Biochem. 2016, 80, 7–12. 10.1080/09168451.2015.1058701. 26103134

[ref18] Liu Y.; Tay J. H. The Essential Role of Hydrodynamic Shear Force in the Formation of Biofilm and Granular Sludge. Water Res. 2002, 36, 1653–1665. 10.1016/S0043-1354(01)00379-7. 12044065

[ref19] Kostenko V.; Salek M. M.; Sattari P.; Martinuzzi R. J. *Staphylococcus Aureus* Biofilm Formation and Tolerance to Antibiotics in Response to Oscillatory Shear Stresses of Physiological Levels. FEMS Immunol. Med. Microbiol. 2010, 59, 421–431. 10.1111/j.1574-695X.2010.00694.x. 20528928

[ref20] Kim M. K.; Ingremeau F.; Zhao A.; Basser B. L.; Stone H. A. Local and global consequences of flow on bacterial quorum sensing. Nat. Microbiol. 2016, 15005. 10.1038/NMICROBIOL.2015.5 PMC5010089 27571752

[ref21] Tsagkari E.; Sloan W. Turbulence accelerates the growth of drinking water biofilms, Bioproc. Biosyst. Eng. 2018, 41 (2), 757–770. 10.1007/s00449-018-1909-0. PMC5958169 29428998

[ref22] Fanesi A.; Lavayssiere M.; Breton C.; Bernard O.; Briandet R.; Lopes F. Shear stress affects the architecture and cohesion of *Chlorella vulgaris* biofilms. Sci. Rep. 2021, 11 (1), 4002 10.1038/s41598-021-83523-3. 33597585 PMC7889892

[ref23] Zabiegaj D.; Hajirasouliha F.; Duilio A.; Guido S.; Caserta S.; Kostoglou M.; Petala M.; Karapantsios T. D.; Trybala A. Wetting/Spreading on Porous Media and on Deformable, Soluble Structured Substrates as a Model System for Studying the Effect of Morphology on Biofilms Wetting and for Assessing Anti-Biofilm Methods. Curr. Opin. Colloid Interface Sci. 2021, 53, 101426 10.1016/j.cocis.2021.101426.

[ref24] Starov V. Surfactant solutions on porous substrates: spreading and imbibition. Adv. Colloid Interface Sci. 2004, 111, 3–27. 10.1016/j.cis.2004.07.007. 15571660

[ref25] Gambaryan-Roisman T. Liquids on porous layers: wetting, imbibition and transport processes. Curr. Opin. Colloid Interface Sci. 2014, 19, 320–335. 10.1016/j.cocis.2014.09.001.

[ref26] Arjmandi-Tash O.; Kovalchuk N. M.; Trybala A.; Kuchin I. V.; Starov V. Kinetics of Wetting and Spreading of Droplets over Various Substrates. Langmuir 2017, 33, 4367–4385. 10.1021/acs.langmuir.6b04094. 28190350

[ref27] Fu F.; Li P.; Wang K.; Wu R. Numerical Simulation of Sessile Droplet Spreading and Penetration on Porous Substrates. Langmuir 2019, 35, 2917–2924. 10.1021/acs.langmuir.8b03472. 30715890

[ref28] Bhattacharjee D.; Nazaripoor H.; Soltannia B.; Ismail M. F.; Sadrzadeh M. An experimental and numerical study of droplet spreading and imbibition on microporous membranes. Colloids Surf., A 2021, 615, 126191 10.1016/j.colsurfa.2021.126191.

[ref29] Chibowski E.; Jurak M.; Holysz L.; Szczes A. Wetting properties of model biological membranes. Curr. Opin. Colloid Interface Sci. 2014, 19, 368–380. 10.1016/j.cocis.2014.03.009.

[ref30] Epstein A. K.; Pokroy B.; Seminara A.; Aizenberg J. Bacterial Biofilm Shows Persistent Resistance to Liquid Wetting and Gas Penetration. Proc. Natl. Acad. Sci. U. S. A. 2011, 108, 995–1000. 10.1073/pnas.1011033108. 21191101 PMC3024672

[ref31] Werb M.; Falcón García C.; Bach N. C.; Grumbein S.; Sieber S. A.; Optiz M.; Lieleg O. Surface topology affects wetting behavior of *Bacillus subtilis* biofilms, npj. Biofilms and Microbiomes 2017, 3, 1. 28649412 10.1038/s41522-017-0018-1 PMC5460217

[ref32] Falcón García C.; Stangl F.; Götz A.; Zhao W.; Sieber S. A.; Opitz M.; Lieleg O. Topographical Alterations Render Bacterial Biofilms Susceptible to Chemical and Mechanical Stress. Biomater. Sci. 2019, 7, 220–32. 10.1039/C8BM00987B. 30426979

[ref33] Thiele U.; Knobloch E. Driven drops on heterogeneous substrates: Onset of sliding motion. Phys. Rev. Lett. 2006, 97, 204501 10.1103/PhysRevLett.97.204501. 17155683

[ref34] Dimitrakopoulos D. Deformation of a droplet adhering to a solid surface in shear flow: onset of interfacial sliding. J. Fluid Mech. 2007, 580, 451–466. 10.1017/S0022112007005721.

[ref35] Beltrame P.; Hanggi P.; Thiele U. Depinning of three-dimensional drops from wettability defects. Europhys. Lett. 2009, 86, 24006 10.1209/0295-5075/86/24006.

[ref36] Varagnolo S.; Ferraro D.; Fantinel P.; Pierno M.; Mistura G.; Amati G.; Biferale L.; Sbragaglia M. Stick-slip sliding of water drops on chemically heterogeneous surfaces. Phys. Rev. Lett. 2013, 111, 066101 10.1103/PhysRevLett.111.066101. 23971591

[ref37] Savva N.; Kalliadasis S. Droplet motion on inclined heterogeneous substrates. J. Fluid Mech. 2013, 725, 462–491. 10.1017/jfm.2013.201.

[ref38] Varagnolo S.; Schiocchet V.; Ferraro D.; Pierno P.; Mistura G.; Sbragaglia M.; Gupta A.; Amati G. Tuning drop motion by chemical patterning of surfaces. Langmuir 2014, 30, 2401–2409. 10.1021/la404502g. 24533817

[ref39] Sbragaglia M.; Biferale L.; Amati G.; Varagnolo S.; Ferraro D.; Mistura G.; Pierno P. Sliding drops across alternating hydrophobic and hydrophilic stripes. Phys. Rev. E 2014, 89, 012406 10.1103/PhysRevE.89.012406. 24580236

[ref40] Engelnkemper S.; Thiele U. The collective behaviour of ensembles of condensing liquid drops on heterogeneous inclined substrates. Europhys. Lett. 2019, 127, 54002 10.1209/0295-5075/127/54002.

[ref41] Busscher H. J.; Bos R.; van der Mei H. C.; Handley P. S. Physicochemistry of microbial adhesion from an overall approach to the limits. In Physical Chemistry of Biological Interfaces; Marcel Dekker AG: New York, 2000; pp 431 450.

[ref42] Bormashenko E. Y. Wetting of Real Surfaces; De Gruyter: Berlin, 2018.

[ref43] Recupido F.; Toscano G.; Tatè R.; Petala M.; Caserta S.; Karapantsios T. D.; Guido S. The Role of Flow in Bacterial Biofilm Morphology and Wetting Properties. Colloids Surf., B 2020, 192, 111047 10.1016/j.colsurfb.2020.111047. 32388030

[ref44] Evgenidis S. P.; Kalić K.; Kostoglou M.; Karapantsios T. D. Kerberos: A three headed centrifugal/tilting device for studying wetting/dewetting under the influence of controlled body forces. Colloids Surf., A Physicochem. Eng. Asp. 2016, 521, 38–48. 10.1016/j.colsurfa.2016.07.079.

[ref45] Ríos-López I.; Evgenidis S.; Kostoglou M.; Zabulis X.; Karapantsios T. D. Effect of initial droplet shape on the tangential force required for spreading and sliding along a solid surface. Colloids Surf., A 2018, 549, 164–173. 10.1016/j.colsurfa.2018.04.004.

[ref46] Rios-Lopez I.; Karamaoynas P.; Zabulis X.; Kostoglou M.; Karapantsios T. D. Image analysis of axisymmetric droplets in wetting experiments: a new tool for the study of 3D droplet geometry and droplet shape reconstruction. Colloids Surf., A 2018, 553, 660–671. 10.1016/j.colsurfa.2018.05.098.

[ref47] Ríos-López I.; Petala M.; Kostoglou M.; Karapantsios T. D. Sessile droplets shape response to complex body forces. Colloids Surf., A 2019, 572, 97–106. 10.1016/j.colsurfa.2019.03.096.

[ref48] Leibniz Institut DSMZ-Deutsche Sammlung von Mikroorganismen und Zellkulturen GmbH; Curators of the DSMZ-4348, (https://www.dsmz.de/collection/catalogue/details/culture/DSM-4358).

[ref49] Potter B. B.; Wimsatt J. Method 415.3, Rev. 1.2, Determination of Total Organic Carbon and Specific UV Absorbance at 254 nm in Source Water and Drinking Water; U.S. Environmental Protection Agency: Washington, DC, 2009, https://cfpub.epa.gov/si/si_public_record_report.cfm?Lab=NERL&dirEntryId=214406&simpleSearch=1&searchAll=415.3.

[ref50] Mintsouli I.; Tsiridis V.; Petala M.; Pliatsikas N.; Rebeyre P.; Darakas E.; Kostoglou M.; Sotiropoulos S.; Karapantsios T. Behavior of Ti-6Al-4 V surfaces after exposure to water disinfected with ionic silver. Appl. Surf. Sci. 2018, 427, 763–770. 10.1016/j.apsusc.2017.08.031.

[ref51] Inouè S.; Spring K. Video Microscopy—The Fundamentals; Plenum Publishing Corp./Springer: New York, 1997.

[ref52] Stevenson K.; Mc Vey A. F.; Clark I. B. N.; Swain P. S.; Pilizota T. General calibration of microbial growth in microplate readers. Sci. Rep. 2016, 6, 38828 10.1038/srep38828. 27958314 PMC5153849

[ref53] Sillankorva S.; Neubauer P.; Azeredo J. *Pseudomonas fluorescens* biofilms subjected to phage phiIBB-PF7A. BMC Biotechnol. 2008, 8, 79 10.1186/1472-6750-8-79. 18954451 PMC2584026

[ref54] Brandon S.; Wachs A.; Marmur A. Simulated contact angle hysteresis of a three-dimensional drop on a chemically heterogeneous surface: a numerical example. J. Colloid Interface Sci. 1997, 191, 110–116. 10.1006/jcis.1997.4912. 9241210

[ref55] Brandon S.; Marmur A. Simulation of contact angle hysteresis on chemically heterogeneous surfaces. J. Colloid Interface Sci. 1996, 183, 351–355. 10.1006/jcis.1996.0556. 8954677

[ref56] Brandon S.; Haimovich N.; Yeger E.; Marmur A. Partial wetting of chemically patterned surfaces: The effect of drop size. J. Colloid Interface Sci. 2003, 263, 237–243. 10.1016/S0021-9797(03)00285-6. 12804908

[ref57] Marmur A. Measures of wettability of solid surfaces. Eur. Phys. J.: Spec. Top. 2011, 197, 193–198. 10.1140/epjst/e2011-01457-4.

