# Supplementary material for: Wetting of Dehydrated Hydrophilic Pseudomonas fluorescens Biofilms under the Action of External Body Forces
Source: Langmuir. 2021 Jul 27;37(37):10890–901. doi: 10.1021/acs.langmuir.1c00528 (PMC8459453; doi:10.1021/acs.langmuir.1c00528)
Supplement: Supplementary file 1 — la1c00528_si_001.pdf [file la1c00528_si_001.pdf]

Wetting of dehydrated hydrophilic *Pseudomonas fluorescens* biofilms under the action of external body forces

Michela Castigliano[a‡], *Federica Recupido*[b‡], *Maria Petala*[c], *Margaritis Kostoglou*[b], *Sergio Caserta*[d+] *and Thodoris D. Karapantsios*[b,*].

a Department of Chemical, Materials and Industrial Engineering (DICMaPi), Piazzale V. Tecchio 80, 80125, Naples (Italy).

b Division of Chemical Technology, School of Chemistry, Aristotle University of Thessaloniki, University Box 116, 54 124 Thessaloniki (Greece).

c Department of Civil Engineering, Aristotle University of Thessaloniki, 54 124 Thessaloniki (Greece).

d CEINGE Advanced Biotechnology, 80145 Naples (Italy).

Supporting information

S1. Glucose concentration in the bacterial suspensions

In Figure S1 the carbon source (Glucose) concentration within bacterial suspensions in the first 24 h after inoculation is shown. Glucose concentration is evaluated according to the sulfuric acid-phenol protocol, reported by Dubois et al.[1]. Briefly, after filtration through a 0.2 μm

membrane, 2 mL samples are mixed with 1 mL of 5 % phenol solution in bi-distilled water and 5 mL of 98 % sulfuric acid solution for 10 min. Next, the resulting solutions are gently homogenized for 30 s and then cooled down for 20 min. Sample OD_{490nm} is then measured. Data are reported as average and standard deviation of three independent measurements.
It is found that glucose concentration rapidly decreases from about 1 g/L to $4.3 \cdot 10^{-4}$ g/L in the first 24 h from inoculation.

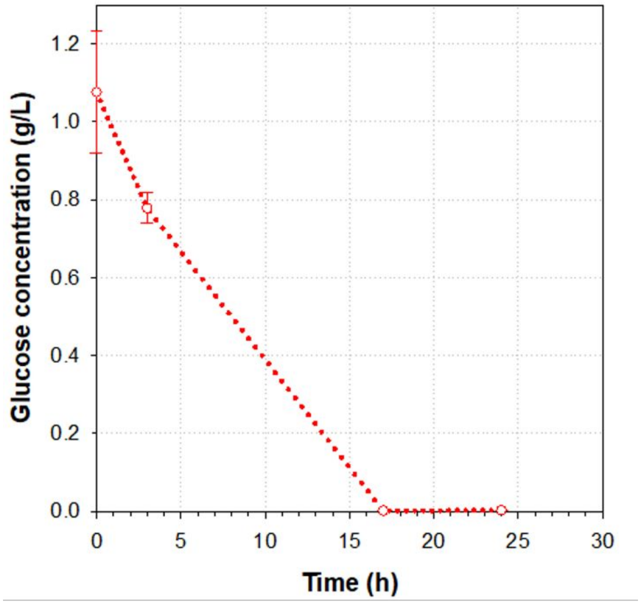

Figure S1. Glucose concentration within bacterial suspensions as function of incubation time. Data are reported as average and standard deviation of three independent measurements.

1) Dubois, M.;.Gilles, K.A.; Hamilton, J.K.;. Rebers, P.A; Smith, F. Colorimetric Method for Determination of Sugars and Related Substances, *Chem*. **1956**, *3*, 350-356.

S2. Repeatability check

To validate the consistency of the experiments, three different repetitions of 10 μL droplet on 3 days-old and 7 days-old biofilms are shown in Figure S2, comparing reproducibility in the measurements of front and rear contact angles and droplet length. Results show that, due to the stochasticity of biofilm structure, wetting properties show quantitative variations among different samples, especially in term of droplet length. However, the qualitative trend observed in the front and rear contact angles as well as in droplet length is similar in all examined conditions, and significantly different from the case of uncoated glass.

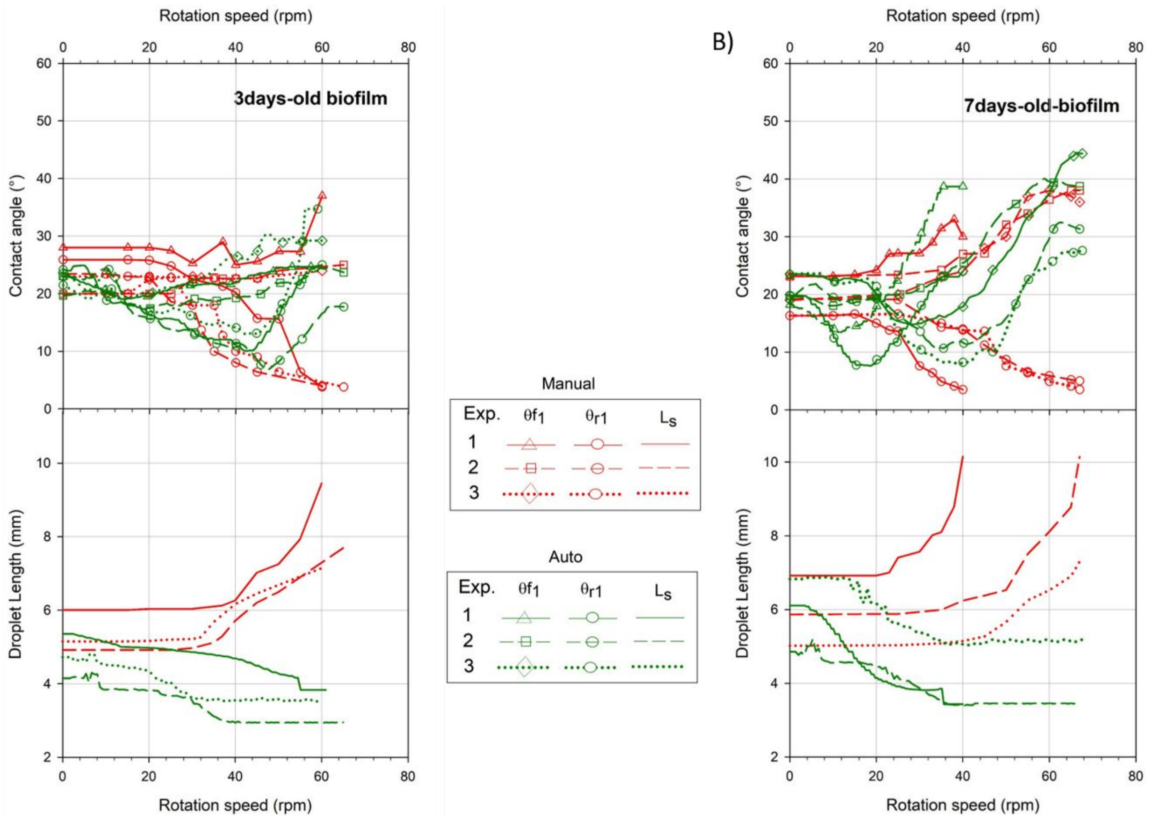

Figure S2. A-B) Front and rear contact angles vs. rotation speed (upper) and droplet length vs. rotation speed (lower) of 10 µL for three different repetitions for 3 days-old biofilm (A) and for 7 days-old biofilms (B), respectively.

S3. Side and top views overlays

In Figure S3 side and top views overlays of 10 µL droplet on 3 days-old biofilm during rotation test, shown in chromatic scale (the black-white version is shown in Figure 8 of the manuscript).

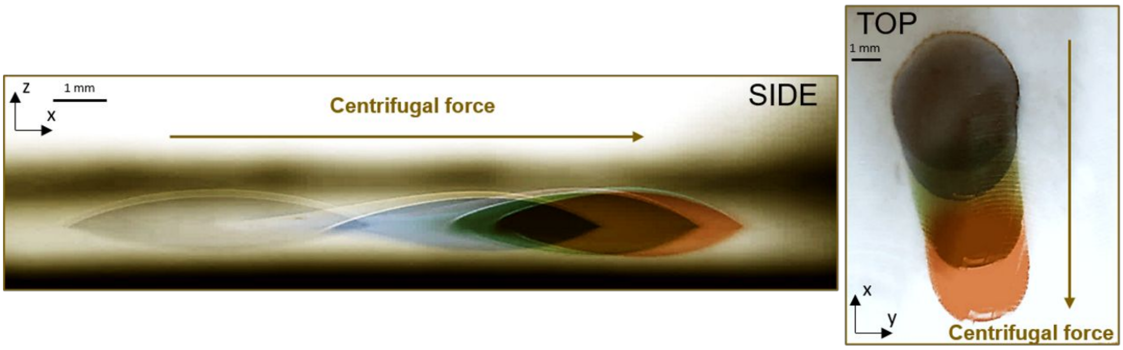

Figure S3: Side view and top view overlays of a 10µL droplet placed onto 3 days-old biofilm during rotation test displayed in chromatic scale.
